# CerebraLux: a low-cost, open-source, wireless probe for optogenetic stimulation

**DOI:** 10.1117/1.NPh.4.4.045001

**Published:** 2017-10-11

**Authors:** Robel Dagnew, Yin-Ying Lin, Jerikko Agatep, Michael Cheng, Andrew Jann, Viola Quach, Michelle Monroe, Ganeev Singh, Ani Minasyan, Joshua Hakimian, Theodore Kee, Jesse Cushman, Wendy Walwyn

**Affiliations:** a University of California, Department of Bioengineering, Los Angeles, California, United States; b University of California, David Geffen School of Medicine, Department of Psychiatry and Biobehavioral Sciences, Los Angeles, California, United States; c University of California, Department of Psychology, Los Angeles, California, United States; d University of California, Brain Research Institute, Los Angeles, California, United States

**Keywords:** wireless optogenetic probe, infrared, open source, optogenetics, low cost

## Abstract

The use of optogenetics to activate or inhibit neurons is an important toolbox for neuroscientists. Several optogenetic devices are in use. These range from wired systems where the optoprobe is physically connected to the light source by a tether, to wireless systems that are remotely controlled. There are advantages and disadvantages of both; the wired systems are lightweight but limit movement due to the tether, and wireless systems allow unrestricted movement but may be heavier than wired systems. Both systems can be expensive to install and use. We have developed a low cost, wireless optogenetic probe, CerebraLux, built from off-the-shelf components. CerebraLux consists of two separable units; an optical component consisting of the baseplate holding the fiber-optic in place and an electronic component consisting of a light-emitting diode, custom-printed circuit board, an infrared receiver, microcontroller, and a rechargeable, lightweight lithium polymer battery. The optical component (0.5 g) is mounted on the head permanently, whereas the electronic component (2.3 g) is removable and is applied for each experiment. We describe the device, provide all designs and specifications, the methods to manufacture and use the device *in vivo*, and demonstrate feasibility in a mouse behavioral paradigm.

## 1 Introduction

Optogenetics is a powerful tool in neuroscience allowing researchers to use light of specific wavelengths to stimulate select neurons with millisecond temporal and spatial precision. This technique comprises two essential components; the expression of an opsin, or light-sensitive ion channel, in specific neurons forming the optic component, and a controllable light-source required to activate the opsin, the electrical component. Opsins, the transducers at the heart of optogenetics, are a class of ion channels expressed on the cell membrane that open to allow ion flow when they receive light of a specific wavelength.^1^ There are many opsin subtypes that have been categorized based on their function and their activating wavelength of light but the most commonly used class of opsins is channelrhodopsin-2 (ChR2). When activated, ChR2 opens and allows the conductance of cations such as sodium, potassium, and calcium across the cell membrane to initiate depolarization and generate action potentials.^2^ The majority of ChR2s are stimulated with the application of blue light of wavelengths ranging from 465 to 473 nm, whereas other variants have been engineered to respond to the red light of higher wavelengths around 620 to 750 nm.^3^ Other opsins have been discovered, generated, or mutated to allow different kinetics of activation, rates of desensitization, responsivity, and multiphoton activation.^4^^,^^5^ Further genetic engineering *in vivo* allows cell-type specific expression of the opsins using the cre-recombinase/loxP system where loxP-flanked opsins are introduced by a virus or genetic engineering into specific cells expressing cre-recombinase. This results in the expression of the opsin in only those cells expressing Cre. Further specificity is provided by the location of the fiber-optic when the optoprobe is implanted. Together, this process allows the activation of specific cells and circuits with millisecond precision and is proving to be an invaluable tool in neuroscience research.

Optogenetics provides neuroscientists the unprecedented ability to investigate the causal relationships between neural activity and behavior in animal models. The wired systems typically consist of an external light source connected via a long fiber-optic cable and commutator, thus tethering the subject to the light source. This imposes some restrictions on a test animal’s movement and limits the application of the experiment. Wireless optogenetic stimulators are commercially available, but they are often too expensive for a standard research budget. One such system available is a head mounted, inductively powered wireless device for mice models with a high starting price for the control unit and further costs per head mount.^6^

On the other hand, there are notable open-source designs available but these require specialized manufacturing facilities. Several designs have been published by the Bruchas and Rogers Research Groups. These use specialized micro- and nanofabrication techniques and require a number of days to manufacture.^7^^,^^8^ Another design by the Poon group utilizes radio-frequency (RF) power to wirelessly power and control a subcutaneously implanted light source but requires manufacturing of a complex RF harvesting resonant cavity. Additionally, operation of the device is restricted to the 21-cm diameter of the RF chamber.^9^ RF has also been used in an earlier design by the Boyden Group, perhaps one of the first wireless devices to be published.^10^ The field has also advanced by incorporating microfluidics to allow a precise delivery of fluids to the neurons being activated^11^ and to assess simultaneous electrical activity.^12^ Another notable design is the infrared (IR)-based stimulator described by Hirase et al.,^13^ which is the most similar available device. While being easier to implement compared to other devices, the need of proprietary software such as LabView to control the parameters of activation for the light-emitting diode (LED) light raises the startup cost. This device also requires more sophisticated manufacturing techniques than CerebraLux, which was designed and built by under-graduate students.

To address these hurdles, we developed CerebraLux: a low-cost, wireless optogenetic probe. The device is designed to be simple to manufacture and assembled without the need for specialized facilities or training. CerebraLux is powered by a light rechargeable lithium polymer (LiPo) battery with sufficient capacity for full behavioral experiments. Most components of the device such as the fiber-optic and electronic surface-mount devices (SMD) are inexpensive and commercially available, whereas custom parts, like the printed circuit board (PCB) and the baseplate, can be manufactured using consumer-grade machines or commissioned from widely available manufacturing services. To demonstrate the feasibility of our system *in vivo*, we implanted our device in the right striatum of mice genetically engineered to express ChR2 crossed with transgenic mice expressing cre-recombinase in dopamine receptor 1 (D1) neurons. This mouse model was chosen as it provides a visually identifiable response to light activation, counter-clockwise turning behavior, and increased motor activity.

For the full realization of the open-source concept, we have included all of our materials, design specifications, computer aided designs (CAD), and computer-aided manufacturing (CAM) files, PCB design, and programming protocols for the microcontroller unit (MCU) and IR communication along with detailed instructions for device assembly and troubleshooting in our compiled CerebraLux manual, included as an Appendix, with this paper. We propose that this information provides a building block to be used by all, either as is or as a building block for further development of the technology.

## 2 Methods

### 2.1 Design and Manufacturing of CerebraLux

#### 2.1.1 Overview of the system

**Fig. 1:**
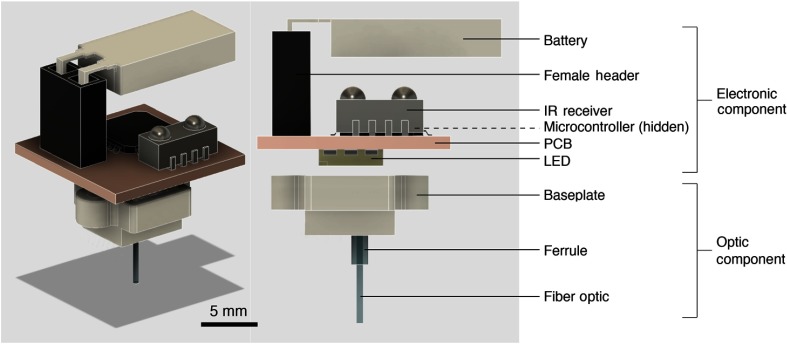
Overview of the CerebraLux optogenetic probe. This schematic shows the two components of the probe: (1) the electronic components consist of the battery, female header, infrared receiver, microcontroller, PCB, and LED. (2) The optic components consist of a baseplate, ferrule, and fiber-optic. The optic component is the only part to be permanently implanted and weighs only 0.5 g, whereas the electronic component weighs 2.3 g and is attached when experiments are run. The electronic and optic components both have magnets that allow for easy attachment and correct alignment between the fiber-optic and LED.

CerebraLux consists of an optic and an electronic component (Fig. 1). The optic component includes a baseplate that holds the fiber-optic and its ferrule. The electronic component includes an MCU, an IR receiver, and an SMD LED soldered on a double-sided PCB with a removable LiPo battery. Only the optic component is permanently housed on the mouse’s head. The two components are aligned and held together using small magnets. This allows the electronic component to be removed when not in use.

#### 2.1.2 Optic component

**Fig. 2:**
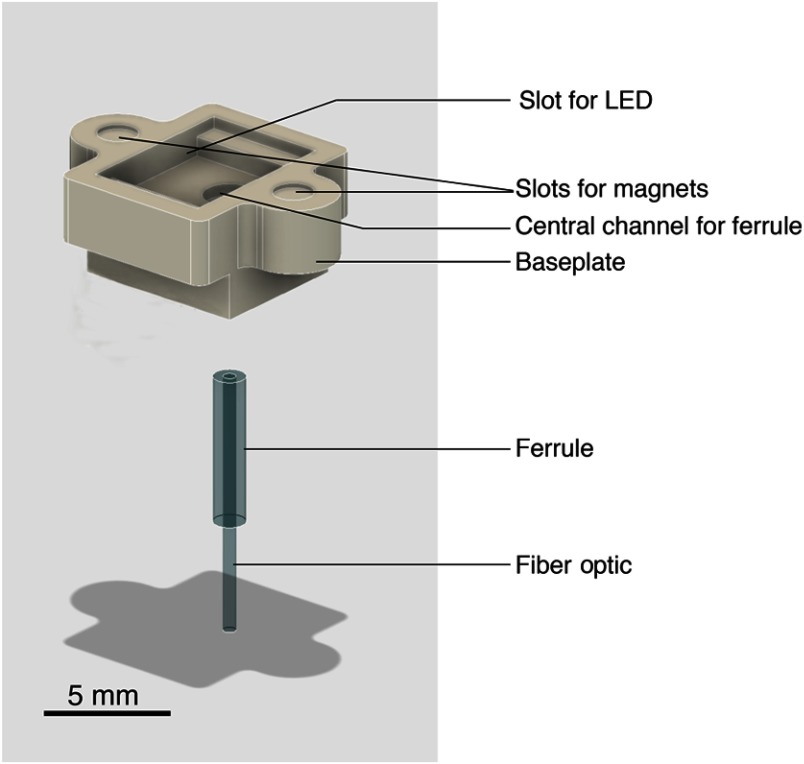
Overview of the optics component on the CerebraLux probe. This schematic shows greater detail of the optic component. The milled baseplate has slots for the magnets, LED, and fiber-optic. The alignment magnets are glued into their respective slots, and the fiber-optic and ferrule are inserted in the central channel of the baseplate. The LED is on the underneath of the PCB and aligns magnetically into the upper slot in the baseplate. This component is inserted into the correct region of the skull using a milled stereotaxic adapter that also has two aligning magnets (Appendix, CerebraLux manual; Sec. A1.5).

An overview of the optic component is shown in Fig. 2. This figure shows the baseplate, made of high-density polyethylene (HDPE), containing the fiber-optic at specific X- and Y-coordinates and two magnets for alignment with the electronic component. The baseplate was designed in Autodesk Fusion 360https://www.autodesk.com/products/fusion-360/students-teachers-educators, an open-source three-dimensional (3-D) design software.^14^ We used this software to generate the CAM toolpaths, and exported the resulting G-code to Otherplan to be milled by an Othermill computer numerical control (CNC) mill (Other Machine Companyhttps://www.bantamtools.com/products/hdpe, Berkeley, California). Step-by-step instructions for the baseplate design, milling, and files for download are available in the CerebraLux manual (Appendix: Secs. A1.1–A1.3) and lab website (Walwyn Labhttp://www.walwynlab.semel.ucla.edu/new-page), respectively. We inserted a 500-μm fiber-optic of specified length based on the coordinates for surgery (Doric Lenses, 480/500-μm diameter, NA 0.63, plastic optical fiber) into a ferrule (Doric Lenses, Zirconia Ferrule OD 1.25 mm). Finally, we inserted the ferrule with the fiber-optic into its channel in the baseplate and glued the magnets (Magnet4us Inc., D0201N40) into their respective slots in the baseplate (Appendix: CerebraLux manual Sec. A1.4).

#### 2.1.3 Electronic component

**Fig. 3:**
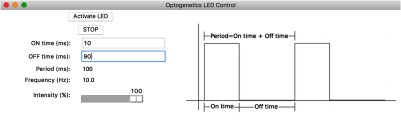
The Python-based GUI. The LED on the PCB is controlled through this Python-based GUI, which can be run on any computer interface. Once installed and the IR controller connected to the computer, the LED is turned on by clicking “activate LED” and turned off by clicking “STOP.” The ON time, OFF time, and intensity can be altered to implement pulse width modulation and light intensity. The GUI also calculates and outputs the period and frequency of the on–off times entered into the GUI.

**Fig. 4:**
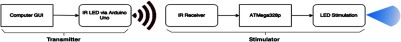
The flowchart demonstrating CerebraLux activation. The computer GUI is connected to an Arduino Uno-controlled IR LED. After pressing “activate LED” on the GUI, the transmitter sends IR pulses to the head-mounted module, where it is received by the photodiode and sent to the ATMega328p microcontroller for processing. The microcontroller then outputs the desired on-time, off-time, and light intensity to the LEDs for stimulating the region of interest in the mouse brain.

**Fig. 5:**
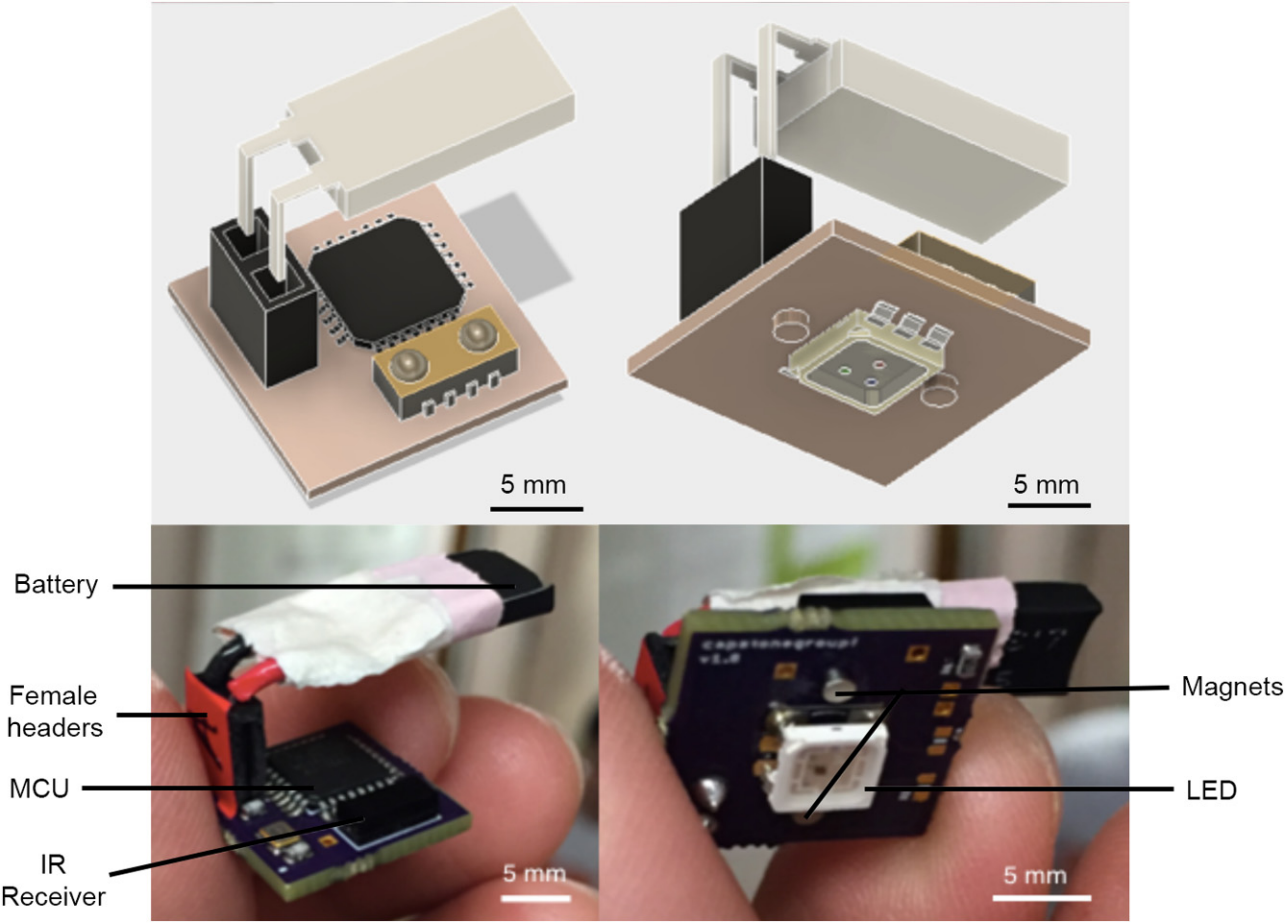
The electronic component of CerebraLux. A fully assembled PCB is shown here. The battery, female headers, MCU, and IR receiver are on the upper side of the PCB and are shown in the left panels with the schematic in the top panel and the device in the lower panel. The magnets and LED are on bottom portion of the PCB. This view is shown in the right panels with the schematic view in the top panel and the device in the lower panel. The module has a footprint of 15×15  mm and, along with female headers and battery, has a weight of 2.3 g. This includes magnets that align with and attach to those on the baseplate of the optic component.

i. *Software*. We used an open-source IR remote library developed by Ken Shirriff for the Arduino platform to send and receive signals, which control the LED. A simple graphical user interface (GUI) was designed to control the stimulator. Figure 3 shows the GUI, which receives the input values for on time, off time, and light intensity, and forwards the information to the device. The GUI communicates serially through a universal serial bus (USB) port to an Arduino Uno (ArduinoBoardUno) to send the corresponding IR LED pulses to the device. We designed the GUI in the Python 2.7 TkInter library, the most widely accessed GUI library. Constructing the GUI in Python instead of proprietary software such as LabVIEW and MATLAB ensures free access and programmability of the interface. By providing our interface as a standalone executable file, researchers can use the interface as is or modify it further to fit their experimental needs. The TkInter library is also well supported, with thorough documentation online in forums and tutorials. The program operates by sending three signals for on time, off time, and light intensity, through the infrared 950-nm LED (SparkFun, Boulder, Colorado) in the form of three IR LED flashes. The IR receiver on the stimulator receives these IR pulses, and the microcontroller processes them to assign the corresponding on–off times and light intensity for the stimulating LED. The order of activation is outlined in Fig. 4. The codes for the sending and receiving protocols in the Arduino interactive development environment (IDE) can be found in the Appendix; CerebraLux manual (Secs. A2.2 and A2.3) while the GUI is downloadable (Walwyn Labhttp://www.walwynlab.semel.ucla.edu/new-page).

ii. *Hardware*. We ported the code onto an ATMega328p MCU using the protocol outlined by Arduino. We then soldered the ATMega328p microcontroller (Atmel, San Jose, California) surface mount IR receiver (Vishay Electronics, Malvern, Pennsylvania), and surface-mount the LED (Kingbright USA, City of Industry, California) onto a custom PCB. We designed the PCB in Cadsoft EAGLE and ordered the PCB to be manufactured by OSHpark. The fully assembled PCB is shown in Fig. 5. We connected a 3.7-V, 20-mAh capacity rechargeable LiPo battery (Tenergy, Fremont, California) to the PCB using female headers (SparkFun, Boulder, Colorado). The LiPo battery chosen weighs under 1 g and fits within a 15×15  mm footprint on the PCB. Finally, we placed two magnets on the bottom side of the PCB that are aligned with those on the baseplate. The final assembly of the electronic component is shown in Fig. 5. Details of all materials used are in the Appendix: CerebraLux manual (Sec. A2.1) and step-by-step instructions to manufacture the PCB are described in the Appendix: CerebraLux manual (Sec. A2.4).

### 2.2 In Vivo Testing of CerebraLux

#### 2.2.1 Surgery to install the CerebraLux baseplate

All animal experiments were conducted with the approval of the UCLA IACUC (OARO). Surgeries were completed in a sterile surgery suite, using a table that allowed 360 deg access and tools that had been sterilized prior to surgery. A Model 1900 Stereotaxic alignment system (Kopf, Tujunga, California) was used for the surgeries. Before use, the Stereotaxic instrument was calibrated using a 40× centering scope (Model 1915, Kopf, Tujunga, California) by centering over the height gauge, identifying, and setting the zero point of reference for the manipulator readout display. The optogenetic mice used were created from two strains: Ai27[RCL-hChR2(H134R)/tdT]-D (JAX #01256) and B6.FVB(Cg)-Tg(Drd1a-cre)EY262Gsat (Mmucd# 030989-UCD) to obtain ChR2 expression in D1 neurons in this Ai27 x D1 Cre mouse line. The mouse was placed in an induction chamber (Patterson Scientific, Foster City, California) and anesthetized with 2.5% isoflurane (Isoflo, Zoetis Inc., Kalamazoo, Michigan). Once breathing slowed, the mouse was removed from the chamber and depth of anesthesia was verified by no response to a toe pinch (stage 3, plane 2 of general anesthesia). Mice were shaved (Wahl, Sterling, Illinois) from eyes to neck along the midline and placed into the nose cone (Model 1923-B Mouse Gas Anesthesia Head Holder, Kopf, Tujunga, California) on a surgical heating pad (Harvard Apparatus, Holliston, Massachusetts). Anesthesia was maintained by 1.5% to 2% isoflurane. 0.5 ml of saline solution (0.9% sodium chloride USP, Hospira, Lake Forest, Illinois) with 1% carprofen (Rimadyl, Zoetis Inc., Kalamazoo, Michigan) was injected subcutaneously to provide fluids and pain relief. Ear-bars were placed so as to secure the skull. The shaved area was cleaned with alternating alcohol and betadine wipes. A 2-cm long incision was made with a #22 scalpel (Miltex, York, Pennsylvania) along the midline from between the eyes to the back of the skull. Brain planarity was verified with an alignment indicator tool (Model 1905, Kopf Instruments, Tujunga, California) on the bregma. Coordinates of bregma were recorded, and the coordinates of probe insertion into the caudate calculated [striatum; A/P(Y)=+0.73  mm, M/L(X)=−2  mm from bregma, and D/V(Z)=−3.5  mm]. The skull was softly scored with a scalpel and the hole drilled at the correct XY-coordinate (Model 1911 Stereotaxic Drilling Unit, Kopf, Tujunga, California). The baseplate with aligned fiber-optic was secured to the stereotaxic frame by the stereotaxic adapter (Appendix: CerebraLux Manual; Sec. A1.5) attached to a cannula holder (Kopf Instruments, model 1766-AP). The baseplate was lowered slowly to the correct D/V(Z) coordinate. Isoflurane was lowered to 1% and a two-part dental cement (Bosworth Trim II, Keystone Industries, Gibbstown, New Jersey) was applied around the baseplate to form a “dome” around the baseplate and to the edge of the scalp. After 5 to 10 min of drying time, the stereotaxic arm and adapter were raised slowly to break the magnetic attachment, leaving the baseplate securely attached to the skull. Isoflurane was then reduced to 0% allowing the mouse to recover from the anesthetic. A triple antibiotic ointment (Perrigo Company plc, Allegan, Michigan) was applied externally around the incision using a Q-tip. After heart rate and breathing increased, the mouse was placed into its home cage preheated on a heating pad (Sunbeam Products Inc., Boca Raton, Florida) until sternal and normal behavior observed. The sham surgery to implant an optoprobe lacking a fiber-optic was completed in the same manner but no hole was drilled in the skull, and the fiber-optic was not inserted.

#### 2.2.2 Behavioral testing

Behavioral testing was completed in a quiet, dark room. A Basler camera model aca1300-60 gc (Ahrensburg, Germany), equipped with a 1/2” Computar IR lens (CBC AMERICAS Corp., Cary, North Carolina) was secured (0.85 m) below the frosted glass panel, in which the subject chambers were placed. The camera was connected to the computer (Windows 10 OS), which ran the behavioral testing software, Ethovision XT 7 (Noldus, Leesburg, Virginia). Tests were conducted in a behavioral arena that was either square (17×13.5×19  cm WxLxH) or circular (17.5 cm in diameter) and data recorded and analyzed by Ethovision (vXT7).

*Testing protocol.* Mice were handled for 3 to 5 days prior to testing. On the day of the test, we first connected the fully charged battery to the PCB, then attached the PCB to the baseplate on the mouse’s head while holding the awake mouse in an open gloved hand. The mouse was then placed into the arena and 5 min of baseline behavior with the light off recorded followed by 5 min of behavior with the light on and the last 5 min with the light off.

*Statistical analysis.* We used Ethovision to measure the number of counter-clockwise and clockwise rotations, total distance traveled, velocity, and time spent mobile in mice implanted with a white-HDPE baseplate (n=3). Video recordings were checked for extraneous tracking and the data exported to and analyzed in Graphpad Prism v7 (Graphpad, La Jolla, California). Ethovision was also used to assess the number of counter-clockwise and clockwise rotations in mice implanted with a black HDPE baseplate (n=2) or the sham mouse implanted with the optoprobe lacking the fiber-optic (n=1) and the data exported to Microsoft Excel (v10) and graphed in Graphpad Prism v7.

## 3 Results

### 3.1 Transmission Range

We first measured the distance that our device was able to consistently send and receive signals and found this to be 1.8 m. This distance was not affected by glass or plastic in the light path. However, we did find that fluorescent lighting interfered with the IR transmission so it is advisable to use low or alternative sources of light.

### 3.2 Stimulator Light Output

**Fig. 6:**
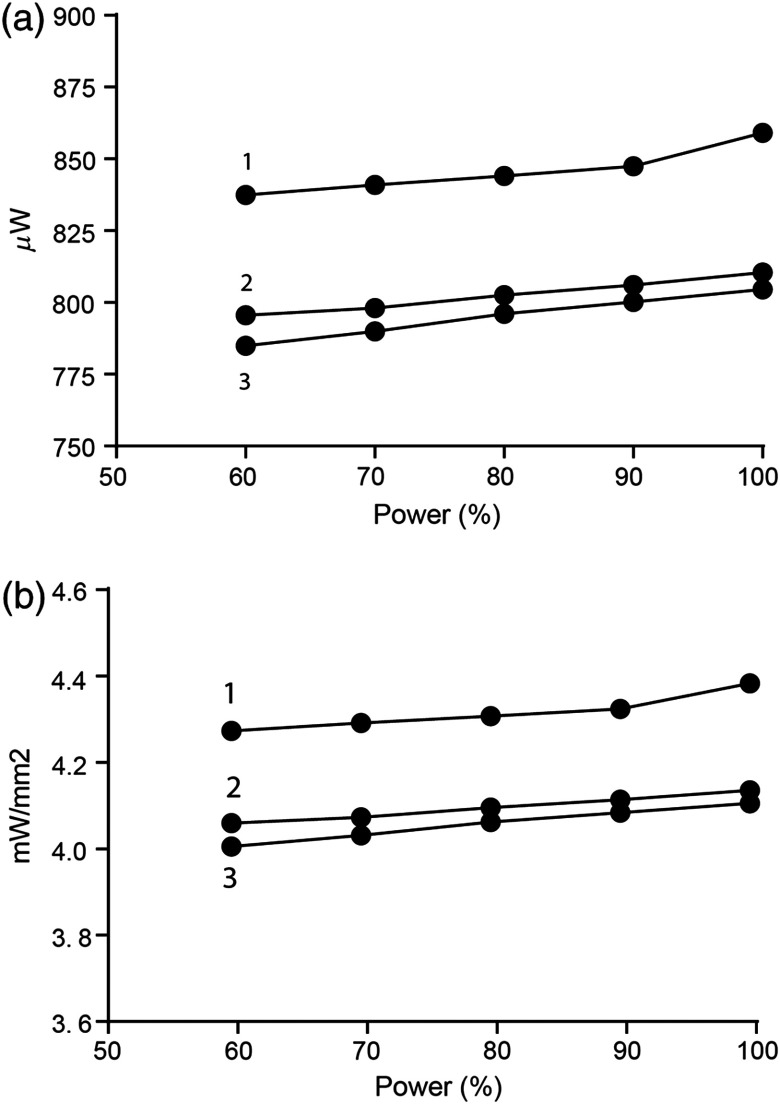
Power output as a function of forward voltage. The effect of varying the forward voltage or intensity (%) on light power output was assessed in the same device connected to three fully charged batteries of different recharge cycles. The data are shown as the total power output (a) and irradiance (b) and show that power output was reduced by 3% if the forward voltage is reduced by 40%. Across all voltages, the battery with fewer recharge cycles (1) had a higher power output compared to batteries with more recharge cycles (2, 3) but all batteries showed the same % reduction with a decrease in forward voltage.

The ability to adjust the light output from the stimulator is critical. Excessive light power may compromise neuron function, whereas light power below the activation threshold will not activate ChR2. The minimum light power needed to activate ChR2 depends on a number of factors including the light attenuation properties of the target brain region, ChR2 expression levels, and the age of the tissue. Typically, 2 to $10\;\mathrm{mW}/{\mathrm{mm}}^{2}$ is considered an acceptable irradiance range to successfully stimulate opsins.^15^ Power can also be manipulated by incorporating pulse-width modulation (PWM) to apply the light at a specific frequency (Hz). A range of frequencies may be used *in vivo* and *in vitro*^16^^–^^19^ but for the tests in this study we used 10 Hz, or 10 ms on and 90 ms off. This is done by changing the “ON” and “OFF” time variables in the GUI (Fig. 3). Power can also be manipulated by changing the forward voltage, shown as the intensity variable in the GUI (Fig. 3). This was assessed *in vitro* by placing a fully assembled device in an integrating sphere (PMD100, Thorlabs, New Jersey) to assess light power output from the tip of the fiber-optic. Furthermore, the performance of rechargeable batteries may be affected by the number of recharge cycles. We, therefore, tested power output using three different batteries that had been recharged once (battery 1), three times (battery 2), or multiple times (battery 3) beforehand. The data are shown as total power output (μW) or irradiance [$\mathrm{mW}/{\mathrm{mm}}^{2}$, Figs. 6(a) and 6(b)]. We found a linear relationship (r=0.98) between the forward voltage and light power output with a peak average output of 825±17  μW or $4.2\pm 0.09\;mW/{\mathrm{mm}}^{2}$. Interestingly, the power output declined by only 3% when the forward voltage was reduced by 40%. Across all voltages, the newer battery (1) had a higher power output compared to the older batteries (2,3), but all batteries showed the same % reduction with decreasing forward voltage. Details of the assembly and use of these batteries can be found in the Appendix: CerebraLux manual (Sec. A2.5).

### 3.3 Stimulator Light Output over a Single Discharge Cycle (Battery Runtime)

**Fig. 7:**
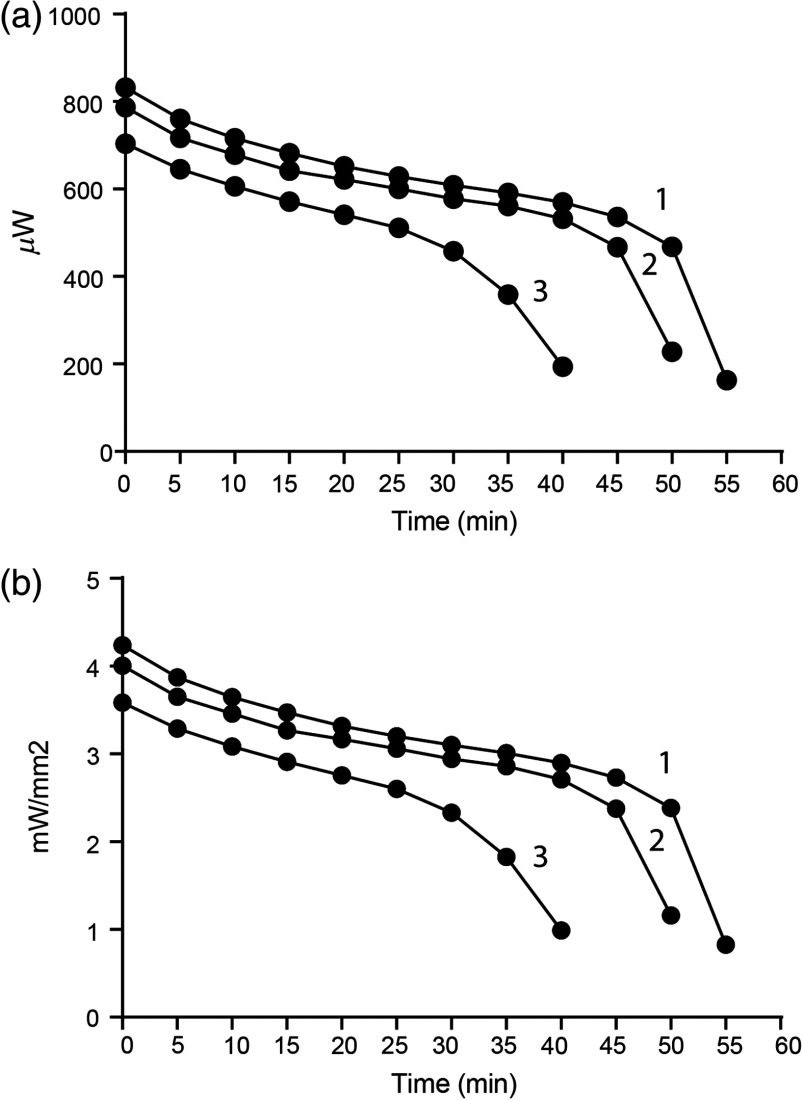
Battery runtime. The lifetime and performance of three batteries was assessed by recording light power output at experimental conditions (10 Hz, 100% forward voltage or intensity) until a threshold of 200  μW was passed. The battery with fewer recharge cycles (1) had a highest initial power output compared to a battery that had recharged 3 (2) or 10+ times (3). During the first 30 min, the power output of all three batteries showed a steady decrease of ∼10% for each 10’ of stimulation that was independent of the initial power output. Thereafter, power output declined but remained above 50% of the initial output for 35 min (3) or 50 min (1, 2).

Well-characterized battery life and power output over a discharge cycle provide critical information for a proposed experimental design. We measured the runtime of the 3.7-V 20 mAh battery tested at a 10-Hz frequency and 100% forward voltage while recording light power output from the fiber-optic tip every 5 min until the battery was not able to maintain the threshold light output of 200  μW. We used the same three batteries as used in the previous test that been recharged once (battery 1), three times (battery 2), or multiple times (battery 3) beforehand. The data show a decline in power output over time (Fig. 7). During the first 30 min, the power output of all three batteries show a steady decrease of ∼10% for each 10 min of stimulation that is independent of the initial power output. Thereafter, power output declined but remained above 50% of the initial output for 35 min (battery 3) or 50 min (batteries 1 and 2). Together, these data (Figs. 6 and 7) show that power output varies relatively little if the forward voltage is reduced, but it is influenced by the number of prior recharge cycles of the battery and the time in use. These data show that it is important to monitor these two parameters during and between experiments.

### 3.4 Testing the Stimulator In Vivo

**Fig. 8:**
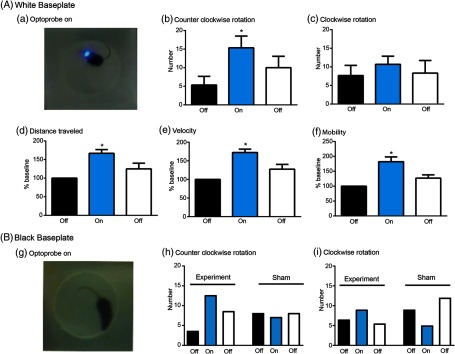
Validation of CerebraLux *in vivo*. (A) White HDPE. Mice (Ai27 x D1-cre, n=3) were implanted with the baseplate, made from white HDPE, containing the fiber-optic (the optic component) and allowed to recover. Shortly before the experiment, the PCB (the electronic component) was attached and mouse placed in the open field for 5 min, the probe was turned on for 5 min (a) and then off for the last 5 min. The behavior was video-tracked and the data exported and analyzed. We found that ChR2 activation in the right striatum (b) increased the number of counter-clockwise rotations, (c) did not alter the number of clockwise rotations, and increased distance traveled (% baseline, d), velocity (% baseline, e), and time spent mobile or % mobility (% baseline, f). *p<0.05 versus the first “OFF” period. (B) Black HDPE. Mice (Ai27 x D1-cre, n=2) were implanted with the baseplate, made from black HDPE, and the same experiment was conducted as in A (experiment). There was a marked reduction in light seen when the probe was turned on (g) but the same relative increase in counter-clockwise, but not clockwise, rotations was observed as in mice implanted with a white HDPE baseplate. A sham mouse (Ai27 × D1 cre), implanted with an optoprobe without the intracerebral fiber-optic, showed no counter-clockwise rotations and a decrease in clockwise rotations when the probe was turned on (h) and (f).

**Fig. 9:**
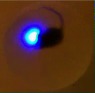
Increased turning when Cerebralux is turned on. This video shows turning behavior in a mouse implanted with a CerebraLux probe. The mouse, with a white HDPE-CerebraLux probe, placed in the right striatum, was placed in an open-field chamber on an elevated, semitransparent, and glass plate. The video, was taken by a camera mounted below the subject and shows turning behavior for 10 s before the optoprobe was turned on, for 5 min when the probe was on and for ∼10  s after stimulation has finished. Note that the view of subject is from below this reversing the direction of rotation and that the speed of the video is increased to a 2× speed throughout (Video 1, MP4, 10.3 MB [URL: http://dx.doi.org/10.1117/1.NPh.4.4.045001.1]).

Activating dopamine 1 (D1) receptors in direct pathway striatal projection neurons increases turning behavior.^20^ Optogenetic activation of these neurons also increases locomotion and velocity.^21^^,^^22^ We proposed that CerebraLux activation of ChR2 in neurons expressing D1 receptors in the right side of the striatum would increase both counter-clockwise rotations and total mobility. This was tested by connecting the electronic component to the *in situ* baseplate, made of white HDPE [Fig. 8(A)], and placing the mouse, from the genetically engineered Ai27 × D1 cre line, in the recording chamber. Details of the operating instructions of the probe and software can be found in the Appendix: CerebraLux manual (Sec. A3). Three mice were used for these tests. After 5 min of basal recording, the optoprobe was turned on (10 Hz, 100% intensity) for 5 min and then off for the final 5 min of the test. Figure 8(Aa) shows the mouse with the optoprobe turned on in the arena used. During this period, counter-clockwise rotations increased [$F_{\left(2,4\right)}=14.19$, p<0.05] when compared with the basal off period [Fig. 8(Ab)] without changing clockwise rotations [$F_{\left(2,4\right)}=4.19$, Fig. 8(Ac)]. Activation of the optoprobe also increased distance traveled [$F_{\left(2,4\right)}=7.367$, p<0.05, Fig. 8(Ad)], velocity [$F_{\left(2,4\right)}=13.13$, p<0.05, Fig. 8(Ae)], and mobility [$F_{\left(2,4\right)}=23.45$, p<0.005, Fig. 8(Af)] versus % basal levels. An example of this behavior is seen in the video (Fig. 9). Together these data show that CerebraLux produces the predicted behavioral outcome, an increase in turning behavior, in mice expressing Ch2R in direct pathway striatonigral neurons.

So as to reduce light scatter, we repeated these tests using a baseplate made of black HDPE in two additional mice of the same genotype [Fig. 8(B)]. This reduced light scatter [shown by the frame taken when the oproptobe was turned on in Figs. 8(Bg) versus 8(Aa)] but did not change the % increase in counter-clockwise rotations during the on versus basal off period [350% versus 348% in mice with black versus white HDPE, respectively, Fig. 8(Bh)]. Turning the optoprobe on also induced a similar % change in clockwise rotations compared with the basal off period; 138% versus 157% in mice with black versus white HDPE, respectively [Fig. 8(Bi)]. In addition, the sham mouse, from the Ai27 × D1 cre line, in which an optoprobe lacking the fiber-optic was implanted, showed no change in counter-clockwise rotations [Fig. 8(Bh)] and a decrease in clockwise rotations [Fig. 8(Bi)] when the probe was turned on. These data show that the use of the black HDPE baseplate did reduce light scatter but did not affect turning behavior resulting from activation of striatal D1 neurons.

## 4 Discussion

This paper provides all the information and methodology required to build an inexpensive wireless optoprobe, CerebraLux. Using these guidelines and specifications, any laboratory interested in optogenetic studies, regardless of budget, will be able to build this lightweight and inexpensive wireless probe. The highlights of CerebraLux are:

### 4.1 Ease of Use

There are several features of the CerebraLux design that make it easy for a researcher to use. The low-profile lightweight baseplate is stable, so it is unlikely to come loose once secured to the skull. The magnets on both the baseplate and PCB allow easy application of the PCB to the mouse. This plug-and-play feature contrasts with wired system optoprobes where anesthesia is often required to connect the mouse. Once in place, the IR-based controller with a transmission range of 1.8 m facilitates complex experimental designs that are not possible using wired or some wireless devices. Once the device has been activated, the mouse can move through mazes or doors allowing further flexibility in experimental designs. Not only is the total light power output adjustable using PWM, the rechargeable battery is also able to sustain 30 min of activation, considerably longer than the typical activation period. Finally, the ability to control CerebraLux through downloadable software on any computer is both easy and inexpensive. The GUI is written in open-source Python to ensure access and programmability by researchers, whereas the IR communication protocol is written in the widely documented Arduino IDE. With all of the implemented features, the total weight of CerebraLux is 2.8 g, of which the mouse permanently carries 0.5 g of the optic component, with the remaining 2.3 g electronic component being detachable. This allows the mouse to move unencumbered during and after experiments. A summary of the features of our device in comparison with similar devices is shown in Table 1.

**Table 1:** Comparison of CerebraLux with other available devices.

	CerebraLux	Cambridge NeuroTech^6^	Hashimoto et al.^13^	Eicom USA	RF Chamber^9^
Size	$0.95\;{\mathrm{cm}}^{3}$	$1.19\;{\mathrm{cm}}^{3}$	14×14 mm	13×18×7 mm	10 to 25 mm^3^
Weight	2.8 g (removable electronics = 2.5 g; permanently implanted optics = 0.3 g)	2.9 g	2.4 g	1.5 g	20 to 50 mg
Range (m)	1.8	4	15	1 (IR remote controller)—3 m (emitter)	0.21 (limited by RF chamber size)
Battery time	35 min (rechargeable)	2 h	10 mAh LiPo 67 min	<1 h	None (RF scavenging)
Fabrication time; difficulty	<2 h; low difficulty	N/A	N/A	1 month lead time	∼11 to 14 days for fabrication; high difficulty (requires microscale surface mount soldering technique and machining of the resonant cavity)
Starting price	$108.10	$9,175 for control unit, headstage, and implant	N/A	$4950 for control unit and receiver	$10,800 Signal generator and power amplifier
Additional costs	$50.50 for each additional headstage (electronics) $52.50 for each additional implant (baseplate and fiber-optic)	$3,000 for each additional headstage, $175 for each additional implant			

Note that only CerebraLux can be manufactured without microscale fabrication techniques. All parts of CerebraLux are available either off the shelf or are easily reproducible using standard manufacturing tools accessible to most laboratories.

### 4.2 Ease of Assembly

The CerebraLux device is designed to be assembled without prior electronic and manufacturing experience. Step-by-step instructions detailing how to manufacture every component of the device are included in the CerebraLux manual. It is also possible to outsource the manufacturing of certain components (e.g., the baseplate) to high-resolution 3-D printing or laser sintering services such as Protolabs. Most of the components, except those that are milled, are commercially available. The baseplate can be machined in 1 h using a low-cost, consumer-grade CNC mill, and the stereotaxic adapter printed in 15 min using a 3-D printer (uPrint SE, Stratasys, Eden Prairie, Minnesota). The PCB board can also be manufactured on the Othermill on a copper board, which is less expensive than OSH Park. Once the PCB is manufactured, researchers can follow the instructions in the CerebraLux manual on soldering all the surface mount components (Sec. A2.4). Manufacturing and assembly of CerebraLux requires a total of 3 h once all the components are attained. In contrast, the cellular-scale stimulator created by the Bruchas research group requires specialized microfabrication facilities and a lengthy production time of two weeks to complete.^7^ CerebraLux’s rapid turnaround time of a day in manufacturing means that design iterations can be developed and tested in a shorter span of time. Additionally, multiple devices can be assembled and implanted at the same time, thus increasing the number of optogenetic studies that a laboratory can conduct within a particular time frame.

### 4.3 Cost Analysis

CerebraLux alleviates the financial barriers to optogenetics technology with its open-source design and low cost. Table 2 shows a price breakdown of all components used in our device and a cost analysis comparing our device with the current standards in the field of optogenetics. Our starting price of $108.10 is considerably less expensive than the Cambridge Neurotech device^6^ or the RF resonant cavity proposed by the Poon group^9^ and other suppliers. It is important to note that this cost analysis does not include the cost of purchasing a CNC mill. However, these are often available on university campuses and, even if outsourced, CerebraLux still remains less expensive than similar commercially available devices.

**Table 2:** Price breakdown of all components used in CerebraLux.

Component	Price
Fiber-optic + fiber-optic ferrule	$50.00
Magnets (8×)	$0.80
Baseplate + baseplate cover (machined)	$0.30
Stereotax holder (3-D printed)	$1.00
SMD LED	$2.50
Ultrathin LiPo battery	$8.50
Microcontroller	$4.00
Microcontroller development board	$5.00
PCB	$2.00
Arduino	$30.00
Resistor, timing crystal, capacitor	$4.00
Total	$108.10

Note: Excluding the Arduino, the fiber-optic and its ferrule holder, all other components are individually priced at under $10 each.

### 4.4 In Vivo Testing

Our behavioral experiments demonstrate proof-of-concept that we are able to target and stimulate opsin-expressing cells in the brain with our low-cost device. As previously mentioned, the appropriate light power needed for an experiment often falls within a narrow range. Our device is able to produce 824±17  μW of power, which, for a 500-μm diameter fiber, corresponds to irradiance values of $4.2\pm 0.1\;\mathrm{mW}/{\mathrm{mm}}^{2}$. Our behavioral testing shows that this is adequate to stimulate ChR2. It must be noted that this is affected by the battery, in particular, the number of recharge cycles. Our data show that a greater number of recharge cycles reduces the maximum light power output and runtime. This reduction can be partially offset by careful soldering of the connections and observing the manufacturer guidelines for care and recharging of the battery. In addition, as these batteries are relatively inexpensive and available, a supply of backup batteries would be advisable.

### 4.5 Future Directions

In providing the complete specifications of CerebraLux, we are providing the foundation, in which this and future iterations of CerebraLux may be built. We have shown that, as is, CerebraLux works *in vivo*. As with any engineering project, there are also future directions and improvements that would enhance and improve this probe. One of these is the modality used to activate the probe. We successfully utilized IR signaling as a medium of communication between the researcher and the stimulator. IR signaling is easy to implement using commercial off-the-shelf components such as IR LEDs and Arduino boards. The ease of IR programmability was also demonstrated by sending signals with an Arduino Uno utilizing popular open-source IR libraries. However, IR requires line-of-sight communication and is affected by ambient light sources, which may restrict the type of behavioral studies that can be done. In future iterations, an approach similar to Hirase et al.^13^ can be adopted using an array of LEDs to transmit the signals from multiple angles, ultimately reducing interference and signal loss. Alternatively, the fragility of IR transmission can be bypassed through use of Bluetooth Low Energy 5.0 technology embedded in microcontrollers; however, this method also presents challenges, such as implementing Bluetooth communication protocols.

Future iterations of our device should also aim to maximize efficiency of the optic assembly. Currently, the LED we used produces 8.1 mW of light power at maximum forward voltage, but 0.824 mW of total power or $4.2\;\mathrm{mW}/{\mathrm{mm}}^{2}$ is transmitted through the fiber-optic at a 10-Hz frequency, a transmission efficiency of 10.6%. It must be noted that our device utilizes direct butt-coupling between the diode and the waveguide with no optical focusing mechanism, explaining the low transmission efficiency. An optical focusing mechanism could be established by lenses and condensers that would collimate the light from the LED before transmission through the fiber-optic and allow the device to be operated in experimental setups with greater light power needs. However, incorporating such focusing elements would also increase the weight and height profile of the device. The baseplate could also be manufactured with higher precision and tolerance to improve alignment between the diode and fiber-optic and to further improve light power transmission. These iterations would increase power output and would also allow a lower voltage to be used, prolonging overall battery life. Another iteration would be to use black HDPE [Fig. 8(B)] for the baseplate so as to minimize light leakage and prevent the mouse from visually reacting to the light.

Our baseplate design can also be altered to target different classes of opsins. By simply changing the location of the ferrule hole in the baseplate during milling, we can align the fiber-optic with a different color diode on the LED. The LED used here has three color settings (i.e., red, green, blue), which can be programmed to allow for stimulation of a wider range of opsin subtypes. A new baseplate design would also be needed to include bilateral probes. This would require multiple LEDs or a single LED with a light splitter being used to stimulate different targets in the brain. A change in the baseplate design may also be needed for different XY-coordinates for different brain regions or for use in other animal models.

One more consideration for a future design is the addition of electrophysiological recording capabilities. To acquire measurements such as temperature and electric signals, our device will require extensive modifications to the device programming, microcontroller selection, and chassis design. However, the addition of this function would require specialized fabrication techniques, which may make the device harder to manufacture and less accessible for all to use.

## 5 Conclusion

We have presented a complete system for optogenetic stimulation in rodents. Our primary objective was to make our device inexpensive and fully open source. We have successfully kept the cost of manufacturing a fully functional and lightweight optogenetic stimulator under $200. We have included our designs and assembly instructions for the neuroscience community to use and/or change as necessary. Thus, our optoprobe strikes a balance between cost, ease of manufacture, and functionality and hope that this will enhance accessibility to the optogenetics toolkit.

## Figures and Tables

**Fig. 10:**
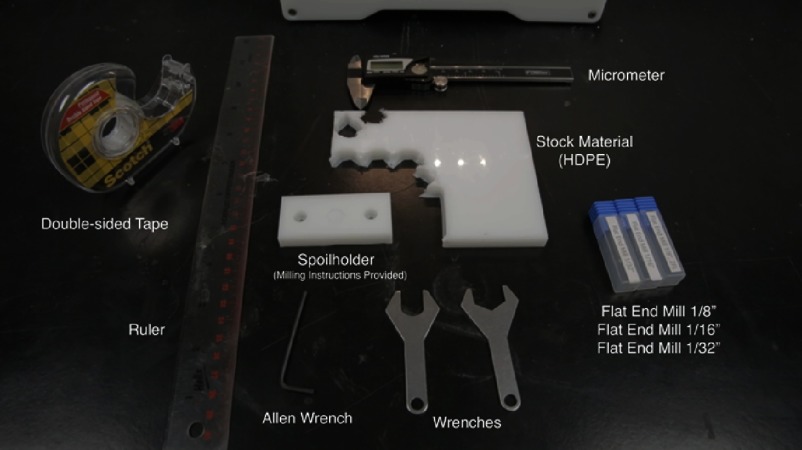
Tools needed for milling.

**Fig. 11:**
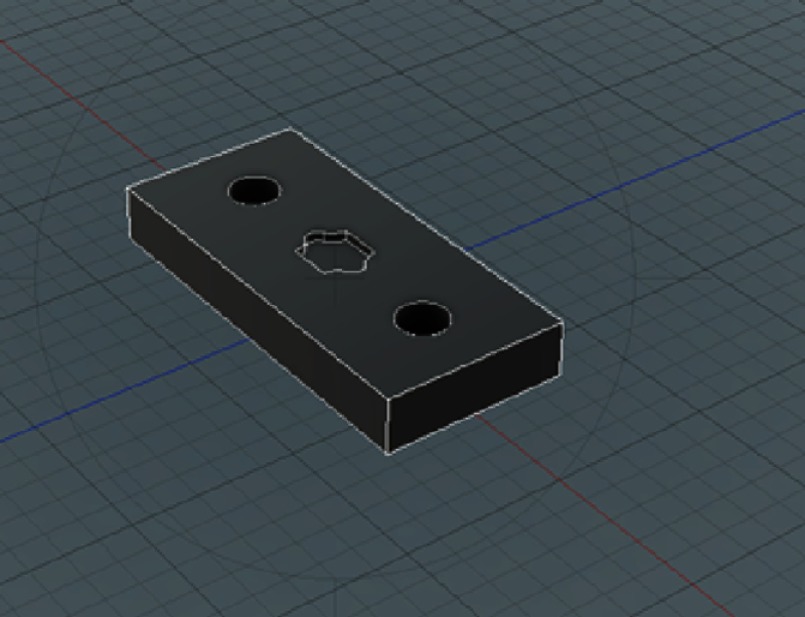
Spoilholder design.

**Fig. 12:**
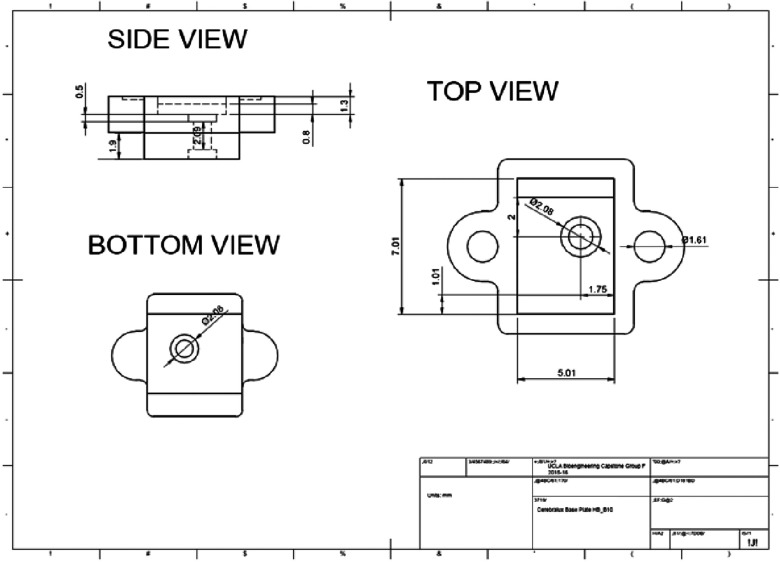
Baseplate dimensions.

**Fig. 13:**
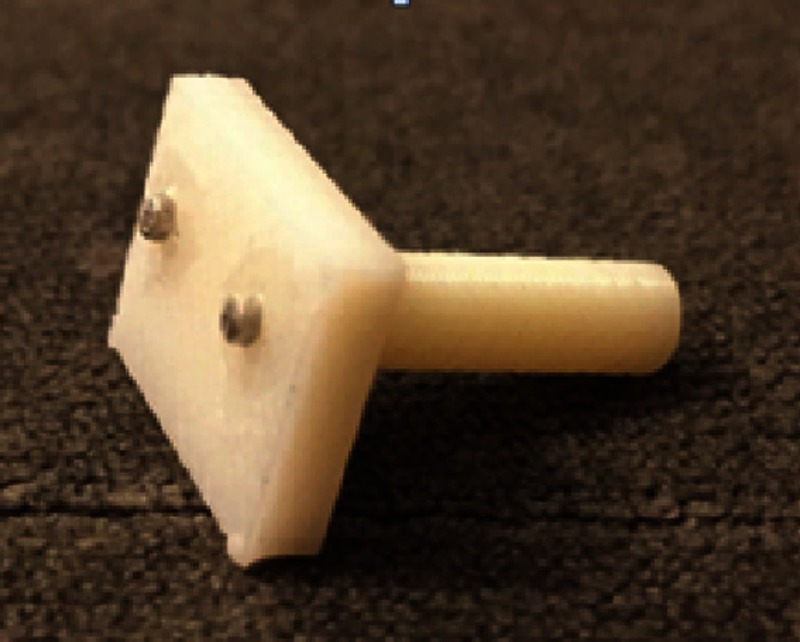
The stereotaxic adapter.

**Fig. 14:**
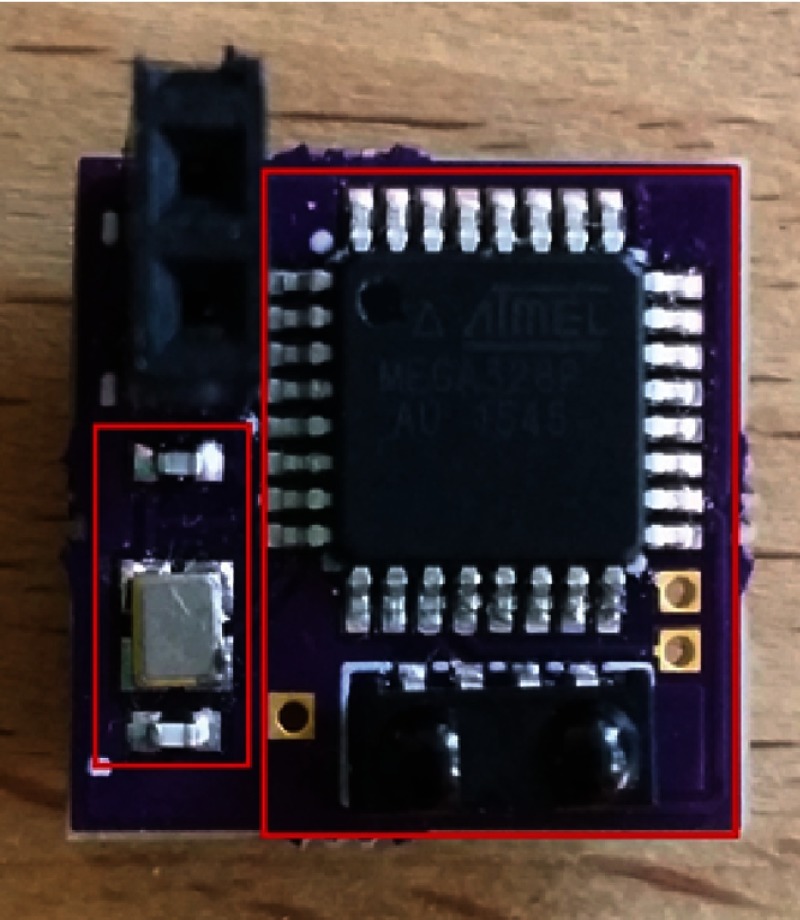
Orientation of SMD components.

**Fig. 15:**
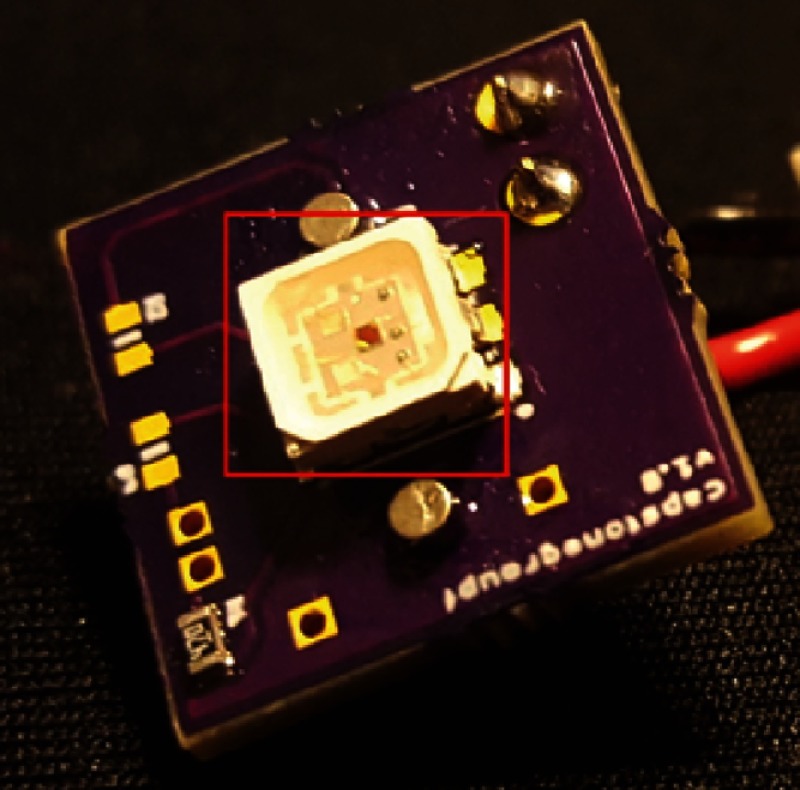
Orientation of LED.

**Fig. 16:**
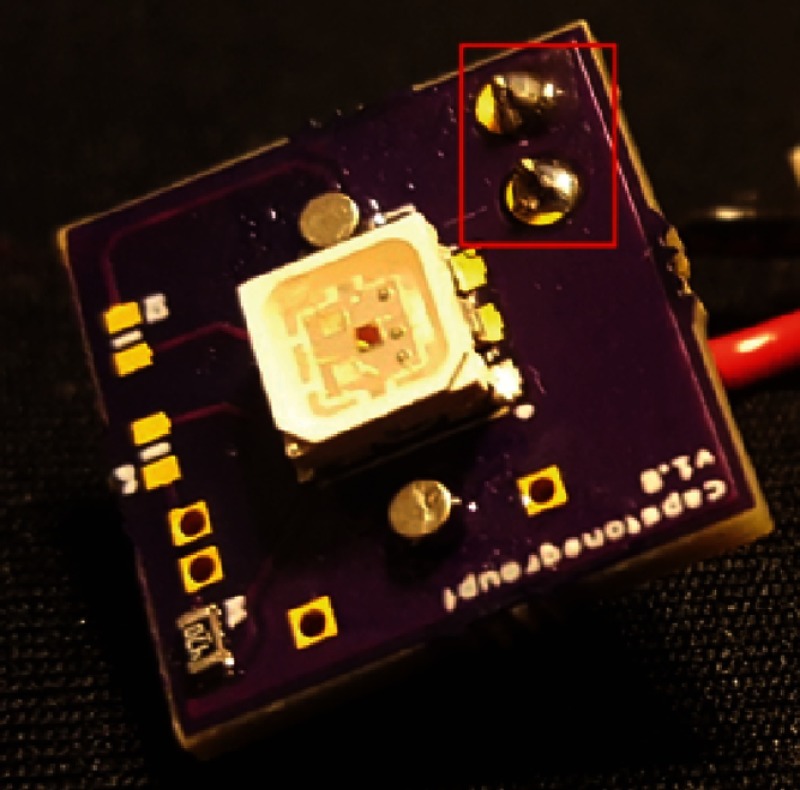
Soldering of female header.

**Fig. 17:**
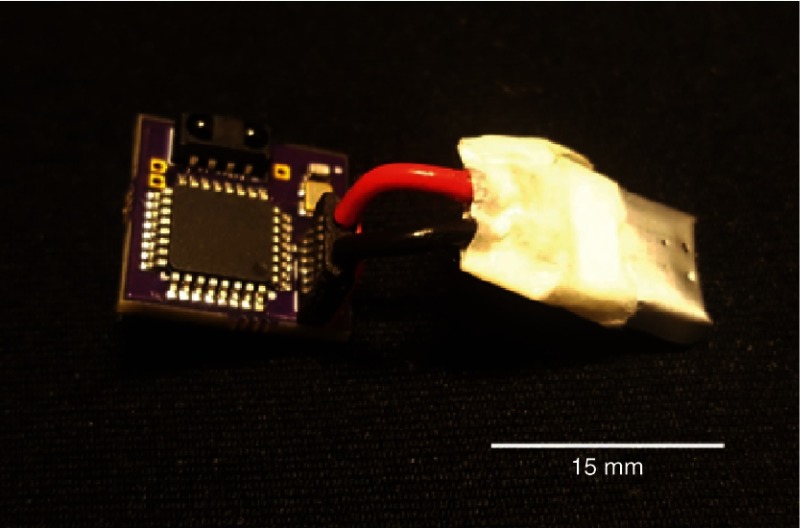
CerebraLux with a connected battery.

**Fig. 18:**
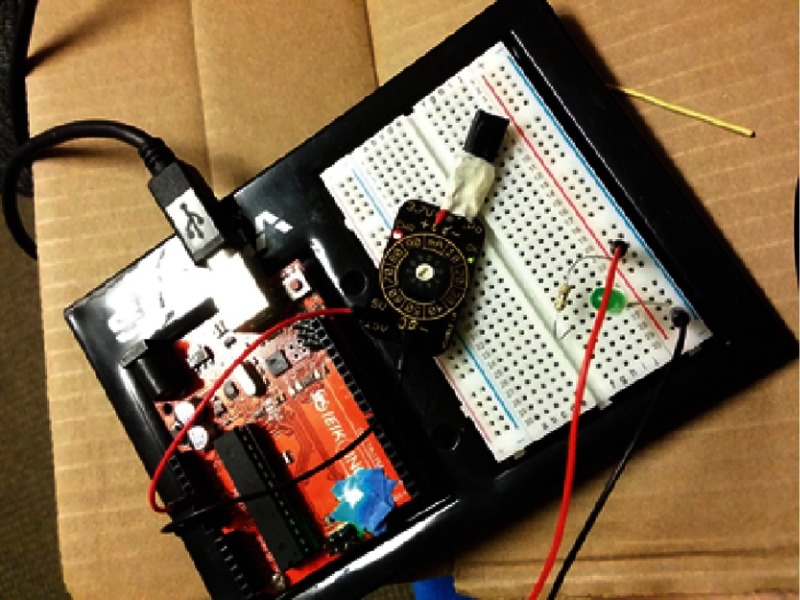
Batter charging circuit.

**Fig. 19:**
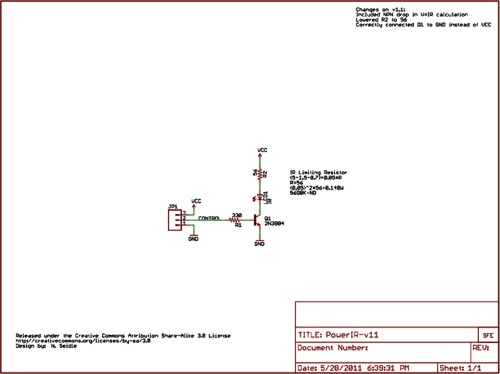
Max IR LED schematic.

## Appendix: Design and Operations Manual

### A1 Baseplate

#### A1.1 Design

The baseplate is the “landing pad” that aligns the LED and its controllers with the fiber-optic. It is permanently attached to the mouse’s head. The top of the baseplate has a rectangular slot for the LED to fit into. Adjacent to the LED’s slot are recesses to accommodate the LED leads and solder joints. The top face of the baseplate also has two cylindrical slots for magnets to be glued into. These two magnets are how the electronic components of CerebraLux are secured onto the baseplate.

The hole for the fiber-optic ferrule is positioned so that when the LED is inserted into the baseplate face-down, the blue diode aligns with the fiber-optic. The proper position for the fiber-optic ferrule channel was determined with digital calipers. The ferrule of the fiber-optic also functions as a surface for the dental cement to adhere to.

#### A1.2 Materials

The baseplate used in CerebraLux was manufactured by CNC milling a block of HDPE. Any soft plastic or metal may be used to create the baseplate, but a lightweight material should be chosen to reduce burden on the test animal.

A 480-μm diameter fiber-optic was used in CerebraLux to maximize the amount of light that the fiber can receive from the LED. Because CerebraLux does not incorporate any light focusing mechanism, a large diameter fiber is recommended to maximize total light power even though light intensity will decrease as a result of the larger surface area.

A 1.27-mm diameter ceramic ferrule was used to house the bare fiber-optic, increasing our ability to polish and handle the fiber-optic. A ferrule is not strictly necessary if the baseplate is modified to accommodate the smaller diameter of the bare fiber-optic instead of a ferrule.

1/16” diameter magnets are used to secure the electronic components to the baseplate. The magnets are glued into their respective slots on the baseplate and on the PCB in alternating polarity to ensure only one way of alignment.

#### A1.3 Milling

Our team used an Othermill tabletop CNC machine to mill the baseplate out of white/black HDPE (HDPEhttps://othermachine.co/store/materials/hdpe/).

Below are instructions for milling the baseplate using the Othermill.

##### A1.3.1 Downloads

a. Download Fusion 360 (Fusion 360) or another CAM software of your choice. Fusion 360 is free for students, educators, and others working in academic institutions. Additionally, it has extra functions such as CAD and animation of 3-D models. Fusion 360 is also the primary software that OtherPlan was designed to interface with.
b. Download OtherPlan (OtherPlanhttp://resources.bantamtools.com/software-download). This is the software used to control the Othermill CNC machine.
c. Download the following 3-D design files:
  Baseplate (Walwyn Labhttp://www.walwynlab.semel.ucla.edu/new-page)
  Spoil holder (Walwyn Labhttp://www.walwynlab.semel.ucla.edu/new-page).

##### A1.3.2 Steps to mill

a. Milling the Spoilholder (Fig. 10); postprocessing the operations in Fusion 360.
  1. First, we will be milling the Spoilholder. This is an auxiliary piece that is used to secure the baseplate when milling its underside.
  2. Open the Spoilholder file in Fusion 360.
  3. Change the workspace to the CAM workspace.
  4. Double click “Setup1.” Go to the “Stock” tab and ensure that the “Height (Z)” dimension matches the thickness of your stock material. Be sure to add ∼0.13  mm to the measured thickness of the stock material to account for the added thickness of the tape used to secure it to the base.
  5. Right-click “Setup1” and click “Generate Toolpath.” This will cause the computer to calculate the paths that the machining tool will follow during the milling process.
  6. Simultaneously select the first three operations (for Macs, press “command” while clicking; for PC’s, press “ctrl” while clicking). Then, right-click the operations and click “Postprocess.”
    Note that you must perform separate postprocessing for each operation that requires a tool change. The first three operations here use a 1/8” diameter flat end mill, whereas the last two operations use a 1/16” diameter flat end mill. As such, separate files must be generated for these two groups.
  7. In the dialogue box, under “Postprocessor,” choose the “othermill.cps—Generic Othermill (Otherplan)” option. Under “Program Number,” enter a descriptive file name that you will be able to recognize when importing into OtherPlan.
    Note: Postprocessing will convert the Fusion 360 CAM operations into a file format that OtherPlan can read.
  8. Repeat steps 6 and 7 to postprocess the last two operations.
b. Milling the SpoilHolder (Fig. 11)—carrying out operations in OtherPlan.
  1. Open OtherPlan.
  2. Plug in the Othermill into your computer via a USB cable and turn the machine on.
  3. Click “Setup Material.” Change material to “HDPE,” and select “Custom Size.” Measure the dimensions of your stock material and input those dimensions into the dialogue box. Note that for the Z-dimension, you should add ∼0.13  mm to account for the added thickness of the tape that you will be using to secure the stock to the spoilboard.
  4. Apply double-sided tape to the underside of the stock material. Cover as much surface area as you can while ensuring that no strips of tape overlap, as this will alter the height of the stock material. Ideally, you should leave some tape hanging over the edge of the stock material as a handle that you can use to remove the stock material after completion of milling.
  5. Firmly press the stock material to the machining bed, taking care to align the corner of the stock material to the corner of the bed.
  6. Click “Import Files.” Select the first group of operations.
  7. Click “Placement” and input the coordinates of where you want the operations to occur on the stock material.
  8. Repeat steps 3 and 4 for the next group of operations. Ensure that both “plans” have the exact same spatial coordinates!
  9. For each operation, click “Add Tool” and select the tool necessary for that operation. The first group of operations uses a 1/8” flat end mill, and the second group uses a 1/16” flat end mill. You can check what tool each operation uses by double-clicking the operation.
  10. Under the “setup” section, click “Set” for tool. Select the tool that you will be using for the current operation. This will initiate the sequence for setting the tool. Otherplan provides instructions to complete this step.
  11. Under the first group of operations, click “Start Cutting.”
  12. Once the first group of operations is finished, “Set” the tool again, and follow the Otherplan instructions to replace the tool bit with the next operation’s tool.
  13. Initiate the second group of operations.
  14. Remove the stock material from the machining bed and use a razor blade to cut the part from the rest of the stock material.
c. Milling the baseplate—milling the top side.
  1. The baseplate has two sets of operations: one for the top of the baseplate (labelled “Top”) and one for the underside of the baseplate (labelled “Bottom”). Postprocess the “Top” baseplate operations as done with the Spoilholder. Be sure to postprocess only consecutive operations that use the same tool bit into the same group.
  2. Open OtherPlan.
  3. Plug in the Othermill into your computer via a USB cable and turn the machine on.
  4. Click “Setup Material.” Change material to “HDPE,” and select “Custom Size.” Measure the dimensions of your stock material and input those dimensions into the dialogue box. Note that for the Z dimension, you should add ∼0.13  mm to account for the added thickness of the tape that you will be using to secure the stock to the spoilboard.
  5. Apply double-sided tape to the underside of the stock material.
    a. Cover as much surface area as you can while ensuring that no strips of tape overlap, as this will alter the height of the stock material. Ideally, you should leave some tape hanging over the edge of the stock material as a handle that you can use to remove the stock material after completion of milling.
  6. Firmly press the stock material to the machining bed, taking care to align the corner of the stock material to the corner of the bed.
  7. Import operation files and input the proper coordinates and tool bits for each operation.
    a. 1/8” and 1/16” bits for the two operations on the top side.
  8. Initiate each cutting operation one at a time, taking care to change tool bits in between each operation.
  9. Once all of the operations are completed, cut the piece away from the rest of the stock material.
d. Milling the baseplate (Fig. 12)—milling the bottom side
  For milling the underside of the baseplate, the baseplate that was manufactured in the previous set of instructions is secured by inserting it upside-down into the Spoilholder. This exposes the underside of the baseplate to cutting operations.
  1. Postprocess the “Bottom” baseplate operations. Be sure to postprocess only consecutive operations that use the same tool bit into the same group.
  2. Remove the spoilboard from the Othermill using an Allen wrench.
  3. In Otherplan, press “Setup Fixturing” and follow the instructions to remove the spoilboard in the software.
  4. Apply a small square of double-sided tape to the top side of the baseplate.
  5. Insert the baseplate, upside-down, into its respective slot on the Spoilholder.
  6. Secure the Spoilholder to the aluminum machining bed with the provided screws and Allen wrench. Make sure to place the Spoilholder so that the position of the fiber-optic channel matches the channel’s position in the CAM file.
  7. In Otherplan, click “Setup material.” Select “HDPE” as the material and enter the following dimensions and coordinates:
    a. X: 29.97 mm
    b. Y: 64.97 mm
    c. Z: 16.31 mm
    d. X-coordinate: 23.48 mm
    e. Y-coordinate: 26.45 mm
    f. Z-coordinate: 0 mm
    g. The Z-dimension of this stock was calculated from the addition of the Spoilholder thickness and the total thickness of the baseplate, with the thickness of their overlap subtracted. The X- and Y-coordinates of the Spoilholder in the Othermill were determined through trial and error.
  8. Import the “Bottom” baseplate operations. By default, the coordinates of the operations are the center of the stock, which is correct for these operations.
  9. Initiate each cutting operation one at a time, taking care to change tool bits in between each operation: 1/8” and 1/32” bits for the two operations on the bottom side.
  10. Once all of the operations are finished, remove the Spoilholder and piece from the Othermill, and detach the completed baseplate from the Spoilholder.

#### A1.4 Assembly

i. Attaching magnets to the baseplate.
  1. Apply Krazy Glue to two magnets. Press the magnets into the two depressions on the top of the baseplate. It is ideal to place the magnets in the baseplate so that they have opposing polarities. This will ensure that the PCB cannot be attached in the wrong orientation.
  2. Using a small applicator, apply glue to the perimeter of the magnets to further secure them to the baseplate. Take care do not get any glue on the top face of the magnets.
  3. Allow the glue to dry.
ii. Attaching magnets to the PCB:
  The magnets are attached to the PCB by a “stamping” method.
  1. Attach a magnet to both of the magnets on the baseplate.
  2. Apply a small spot of Krazy Glue to the faces of the second pair of magnets.
  3. Insert the LED of the PCB into the square slot of the baseplate. Take care to ensure that the LED is in the correct orientation when you insert it. The blue diode of the LED must be aligned with the fiber-optic channel.
  4. Allow the glue to dry.
  5. Detach the electronic components from the baseplate. The magnets should remain “stamped” to the PCB.

#### A1.5 Stereotaxic adapter

To assist in implanting the device, a stereotaxic adapter can be designed and 3-D printed (Fig. 13). The stereotaxic adapter attaches to the baseplate through magnets (shown below) and fits into a cannula holder available from Kopf (Kopf, #1766). Our adapter CAD design is available for download at the Walwyn Labhttp://www.walwynlab.semel.ucla.edu/new-page website.

### A2 Electronics

#### A2.1 Materials

*Custom PCB*

• PCB schematic files (Walwyn Labhttp://www.walwynlab.semel.ucla.edu/new-page)
  ○ Upload and send to OSHpark.com or another PCB manufacturer for production

*IR transmitter and receiver modules*

• Infrared 950-nm LED (SparkFun Part No. COM-09349)
• Infrared receiver (Digikey Part No. TSOP75438TTCT-ND; Vishay Electronics Part No. TSOP75438TT)
• Surface mount 20-Ω resistor (Digikey Part No. AC0402JR-0720RL-ND; Yageo Part No. AC0402JR-0720RL)
• ATMega328p-AU microcontroller (Digikey Part No. ATMega328p-AU-ND; Atmel Part No. ATMega328p-AU)
• Female headers (SparkFun Part No. PRT-00115)
• 2× Arduino Uno
  ○ Remove the microcontroller on one of them
• Full-Color Surface Mount LED Lamp (Kingbright USA Part No. AAAF5051-04)
• Max IR LED schematic 1
  ○ Transistor (Digikey Part No. 2N3904-APCT-ND; Micro Commercial Co. Part No. 2N3904-AP)
  ○ 56-Ω Resistor (Digikey Part No. S56HCT-ND; Stackpole Electronics Part No. CFM12JT56R0)
  ○ 330-Ω Resistor (Digikey Part No. S330HCT-ND; Stackpole Electronics Part No. CFM12JT330R)

*Battery and Charger*

• 20-mAh lithium-ion polymer rechargeable battery (All batteryhttp://www.all-battery.com/Tenergy3.7V19mAhLIPOBattery-30544-0.aspx, PP031012)
• Single cell low current LiPo charger (Leo Bodnarhttp://www.leobodnar.com/shop/index.php?main_page=product_info&products_id=215 electronics)

*Microcontroller programming*

○ 1×16−MHz crystal (Sparkfun Part No. COM-00536)
○ 1×10−k resistor (through-hole) (Sparkfun Part No. COM-11508)
○ 2×18- to 22-pF ceramic capacitors (through-hole) (Sparkfun Part No. COM-085)
○ Jumper wires (Sparkfun Part No. PRT-13870)
○ Breadboard (Sparkfun Part No. PRT-00112)

• 32-pin TQFP adapter
  ○ For easy (but expensive) ATMega328p-AU programming, use this adapter (http://www.digikey.com/product-detail/en/xeltek/SA636/415-1028-ND/970413)
  ○ For more difficult (but significantly cheaper) ATMega328p-AU programming, use this adapter (http://www.digikey.com/product-detail/en/adafruit-industries-llc/1163/1528-1065-ND/5022794)

*Soldering*

• Solder (SparkFun Part No. TOL-09325)
• Soldering iron (Digikey Part No. WES51-120V-ND)
• Solder paste (Digikey Part No. SMD291AX-ND)
• Solder flux (Digikey Part No. SMD291NL-ND)
• Soldering heat gun (Amazon.comhttps://www.amazon.com/WEP-858D-Soldering-Station-Suitable/dp/B0055B6NGE, WEP 858D)

#### A2.2 Downloads

*Arduino IDE 1.6+*: https://www.arduino.cc/en/Main/Software

To use IRremote library:

1. Download the. ZIP file from https://github.com/z3t0/Arduino-IRremote.
2. Move the extracted “IRremote” folder to your libraries directory.
3. Delete the RobotIRremote library in Arduino.
  • Windows: Applications/Arduino/Content/Java/libraries/delete RobotIRremote folder
  • Mac: Finder/Applications/Right-click on Arduino/Show package contents/Content/Java/libraries/delete RobotIRremote folder
4. Make sure to delete Arduino_Root/libraries/RobotIRremote (Arduino_Root refers to the install directory of Arduino). The library RobotIRremote has similar definitions to IRremote and causes errors.

*Python IDE 2.7+ (NOT Python 3)*: https://www.python.org/downloads/

*Pyserial*: http://pyserial.readthedocs.org/en/latest/pyserial.html

• Windows: Open “Command Terminal”/Type *cd Python27*/Type *cd scripts*/Type *pip install pyserial*
• Mac/Linux: Type *pip install pyserial* into command line/terminal

*Python Imaging Library (PIL)*: http://pillow.readthedocs.org/en/3.0.x/installation.html

• Windows: Open “Command Terminal”/Type *cd Python27*/Type *cd scripts*/Type *pip install Pillow*
• Mac/Linux: Type *pip install Pillow* into command line/terminal

*Source code*

1. GUI (download from Walwyn Labhttp://www.walwynlab.semel.ucla.edu/new-page)
2. IR Sending Protocol Table 3: Save this code in the Arduino IDE as IR_LEDsend.ino in a folder named IR_LEDsend anywhere on the drive.
3. IR Receiving Protocol Table 4: Save this code in the Arduino IDE as IR_test.ino in a folder named IR_test anywhere on the drive.

**Table 3:** IR Sending Protocol.

/*
* IRremote: IRsendDemo - demonstrates sending IR codes with IRsend
* An IR LED must be connected to Arduino PWM pin 3.
* Version 0.1 July, 2009
* Copyright 2009 Ken Shirriff
* http://arcfn.com
*/

#include <IRremote.h>
IRsend irsend;
//GUI variables
int mydata;
int input[3];
int zeroSignal = 777;
int numberOfInputs = sizeof(input)/sizeof(int);
int i=0;
void setup() {
Serial.begin(9600);
Serial.setTimeout(100);
}
void loop() {
if (Serial.available()>0) {// when data is received through the Serial port, read data
mydata = Serial.parseInt(); // reads integers until a noninteger, nonnegative sign
if (mydata > 0) {
if (i < numberOfInputs - 1) {
input[i] = mydata;
i++;
}
else if (i == numberOfInputs - 1) {
input[i] = mydata;
for(i=0;i<numberOfInputs;i++) {
Serial.println(input[i]);
irsend.sendSony(input[i], 16); //inputs floats, number of bits
delay(150);
}
i=0;
}
else {
Serial.println(“It’s a trap”);
}
}
}
}
// thisString = String(i, HEX);
// Serial.println(thisString);
//for (int i = 0; i < 1; i++) {
// irsend.sendSony(1000, 12);
// delay(40);
// }
// delay(2000); //2 second delay between each signal burst
// for (int i = 0; i < 1; i++) {
// irsend.sendSony(0xa90, 12);
// delay(40);
// }
// delay(2000); //5 second delay between each signal burst
//}

**Table 4:** IR Receiving Protocol.

/*
IRremote: IRrecvDemo - demonstrates receiving IR codes with IRrecv
An IR detector/demodulator must be connected to the receivedInputs RECV_PIN.
Version 0.1 July, 2009
Copyright 2009 Ken Shirriff
http://arcfn.com
*/

#include <IRremote.h>
int RECV_PIN = 7;
int BAD_PIN = 8;
int LED_PIN = 9;
int receivedInputs[2] = {0,0};
int numberOfReceivedInputs = sizeof(receivedInputs)/sizeof(int);
int pwm = 255;
float intensity;
int zeroSignal = 777;
int ONtime = 0;
int OFFtime = 10;
int i=0;
String resultsString;
int resultsInt;
IRrecv irrecv(RECV_PIN);
decode_results results;
void setup()
{
pinMode(BAD_PIN, INPUT);
pinMode(LED_PIN, OUTPUT);
// digitalWrite(BAD_PIN, HIGH);
Serial.begin(9600);
irrecv.enableIRIn(); // Start the receiver
}
void loop() {
if (irrecv.decode(&results)) {
resultsString = String(results.value);
resultsInt = resultsString.toInt();
irrecv.resume(); // Receive the next value
if (resultsInt < 1000) {
if (i < numberOfReceivedInputs - 1) {
if (resultsInt == zeroSignal) {
resultsInt = 0;
receivedInputs[i] = resultsInt;
i++;
}
else {
receivedInputs[i] = resultsInt;
i++;
}
}
else if (i == numberOfReceivedInputs - 1) {
if (resultsInt == zeroSignal) {
resultsInt = 0;
receivedInputs[i] = resultsInt;
i++;
}
else {
receivedInputs[i] = resultsInt;
}
ONtime = receivedInputs[0];
OFFtime = receivedInputs[1];
Serial.print(“ON time:”);
Serial.println(ONtime);
Serial.print(“OFF time:”);
Serial.println(OFFtime);
i=0;
}
}
else if (resultsInt > 1000) {
intensity = resultsInt/100;
intensity = intensity/100;
pwm = 255 * intensity;
Serial.print("Intensity: ");
Serial.println(pwm);
}
}
else {
if (receivedInputs[0] == 0 && receivedInputs[1] == 0) {
analogWrite(LED_PIN, LOW);
//turn off LED and do nothing
}
else if (receivedInputs[0] == 0 && receivedInputs[1] != 0) {
analogWrite(LED_PIN, LOW);
//turn off LED and do nothing
}
else if (receivedInputs[0] != 0 && receivedInputs[1] == 0) {
analogWrite(LED_PIN, pwm);
}
else {
analogWrite(LED_PIN, pwm);
delay(ONtime);
analogWrite(LED_PIN, LOW);
delay(OFFtime);
}
}
}

#### A2.3 Programming the MCU

1. Make sure that the Arduino, Python, and additional libraries are installed as shown in the previous tutorial.
2. Arduino Uno pinout (http://foros.giltesa.com/otros/arduino/fc/docs/pinout/uno.jpg).
3. ATMega328p-AU pinout (http://i.lnwfile.com/_/i/_raw/6z/8a/x0.png).
4. The microcontroller on an Arduino Uno is the ATMega328p-PU (or breadboard version), whereas the one that will be used is the ATMega328p-AU (surface-mount version).
5. You need to first read this Arduino HowTo: https://www.arduino.cc/en/Tutorial/ArduinoToBreadboard.
  a. The procedure to program the MCU is the same as in this tutorial, but you will be using an ATMega328p-AU with 32-pin TQFP adapter instead of an ATMega328p-PU.
    i. If you are using the easy method, then you will need to apply some pressure and/or tape onto the microcontroller so the leads make a solid connection with the adapter.
  b. Follow everything in the procedure listed in this website (burning the Arduino bootloader then uploading the IR_test), but instead use the ATMega328p-AU with 32-pin TQFP adapter (the pinouts for both microcontrollers are included to accurately identify numbering between the two).
  c. Once this stage is complete, you should have a ATMega328p-AU with the uploaded code.

#### A2.4 Building the PCB

1. Before beginning, watch this tutorial for surface mount soldering and hand soldering, respectively: https://www.youtube.com/watch?v=0XENpPtisnM, https://www.youtube.com/watch?v=f95i88OSWB4.
2. Mix solder paste and flux in a 1∶1 ratio with a toothpick or small applicator.
3. Apply the mixture onto the metal pads of the board within the red squares shown above (Fig. 14).
4. Place components on parts in the same orientation as shown above (Fig. 14).
  a. Use the ATMega328p-AU with uploaded code!
5. Use a solder heat gun to apply heat to the components until the solder fluxes onto the pads (the solder paste should turn from a dull gray to a shiny silver when the process is successful). In this method with the heat gun, start out high on the board and slowly make your way down, moving the gun in a circular fashion to heat up the entire board.
6. Repeat steps 3 to 5 for the bottom of the board on the metal pads within the red square (Fig. 15).
  a. Place the LED in the orientation shown (with cut edges facing the *capstonegroupf* label).
7. For this next step, you will be soldering two of the female headers into the holes within the red box shown above (Fig. 16).
8. Glue the magnets on the board. Remember, you will need to match the polarity of the magnets on the baseplate.

#### A2.5 Battery

i. Preparing and connecting the battery.
  1. Obtain batteries (Allbatteries: Tenergy, PP031012).
  2. Using tweezers, solder the red wire to the positive tab and the black wire to the negative tab. Make sure to use higher gauge/small diameter wires to help reduce strain on the tabs on the battery. Once soldered, make sure to never let the wires touch. Touching these wires together will create a short circuit and will cause the battery to dangerously overheat.
  3. Wrap the tabs with tape for a more secure connection and to prevent accidental short circuiting on that end.
  4. Connect the battery to the PCB as shown in Fig. 17. The positive terminal is closer to the IR receiver and the negative terminal is further away. Make sure the red wire goes into the positive terminal and the black wire goes to the negative terminal. Otherwise, the battery will overheat.
    Note: It may be possible to use connecting wires of smaller diameter to reduce.
ii. Charging the battery.
  1. Make sure you set the charger at or below the rating of the battery (e.g., 10 or 20 mA).
  2. Connect the charger to an Arduino with wires. The positive terminal (red wire) connects to a 5-V terminal on the Arduino. The negative terminal (black wire) connects to a ground terminal on the Arduino.
  3. Connect the battery on the other end using its soldered wires: red to positive and black to negative (Fig. 18). There should be a red light indicating a charging battery and a green light indicating a charged battery.
iii. Operations Guide.
  1. Assemble the Max IR LED circuit according to the schematic shown below in Fig. 19.
  2. Connect pin 3 on the Arduino Uno (w/microcontroller) to the Max IR LED circuit.
  3. Connect Arduino Uno (w/microcontroller) to the computer.
  4. Upload IR_LED send code to the Arduino Uno.
  5. Open up the Python GUI code.
  6. Go to the Run tab and run module.
  7. The GUI should open; input numbers for the on/off times and specify intensity.

## References

[1] Deisseroth K., “Optogenetics: 10 years of microbial opsins in neuroscience,” Nat. Neurosci. 18, 1213–1225 (2015). NANEFN 1097-6256 10.1038/nn.4091 26308982 PMC4790845

[2] Nagel G. et al., “Channelrhodopsin-1: a light-gated proton channel in green algae,” Science 296, 2395–2398 (2002). SCIEAS 0036-8075 10.1126/science.1072068 12089443

[3] Lin J. Y. et al., “ReaChR: a red-shifted variant of channelrhodopsin enables deep transcranial optogenetic excitation,” Nat. Neurosci. 16, 1499–1508 (2013). NANEFN 1097-6256 10.1038/nn.3502 23995068 PMC3793847

[4] Brinks D. et al., “Painting with rainbows: patterning light in space, time, and wavelength for multiphoton optogenetic sensing and control,” Acc. Chem. Res. 49, 2518–2526 (2016). ACHRE4 0001-4842 10.1021/acs.accounts.6b00415 27786461

[5] Wietek J. Prigge M., “Enhancing channelrhodopsins: an overview,” Methods Mol. Biol. 1408, 141–165 (2016). 10.1007/978-1-4939-3512-3_10 26965121

[6] Rossi M. A. et al., “A wirelessly controlled implantable LED system for deep brain optogenetic stimulation,” Front. Integr. Neurosci. 9, 8 (2015). 10.3389/fnint.2015.00008 25713516 PMC4322607

[7] Kim T. I. et al., “Injectable, cellular-scale optoelectronics with applications for wireless optogenetics,” Science 340, 211–216 (2013). SCIEAS 0036-8075 10.1126/science.1232437 23580530 PMC3769938

[8] Shin G. et al., “Flexible near-field wireless optoelectronics as subdermal implants for broad applications in optogenetics,” Neuron 93, 509–521.e3 (2017). NERNET 0896-6273 10.1016/j.neuron.2016.12.031 28132830 PMC5377903

[9] Montgomery K. L. et al., “Wirelessly powered, fully internal optogenetics for brain, spinal and peripheral circuits in mice,” Nat. Methods 12, 969–974 (2015).1548-7091 10.1038/nmeth.3536 26280330 PMC5507210

[10] Wentz C. T. et al., “A wirelessly powered and controlled device for optical neural control of freely-behaving animals,” J. Neural Eng. 8, 046021 (2011).1741-2560 10.1088/1741-2560/8/4/046021 21701058 PMC3151576

[11] McCall J. G. et al., “Preparation and implementation of optofluidic neural probes for in vivo wireless pharmacology and optogenetics,” Nat. Protoc. 12, 219–237 (2017).1754-2189 10.1038/nprot.2016.155 28055036

[12] Gagnon-Turcotte G. et al., “A wireless headstage for combined optogenetics and multichannel electrophysiological recording,” IEEE Trans. Biomed. Circuits Syst. 11, 1–14 (2017). 10.1109/TBCAS.2016.2547864 27337721

[13] Hashimoto M. et al., “Programmable wireless light-emitting diode stimulator for chronic stimulation of optogenetic molecules in freely moving mice,” Neurophotonics 1, 011002 (2014). 10.1117/1.NPh.1.1.011002 26157963 PMC4478966

[14] Fusion 360 free 3D CAD/CAM design software for students, educators, and academic institutions Autodesk, https://www.autodesk.com/products/fusion-360/students-teachers-educators.

[15] Mattis J. et al., “Principles for applying optogenetic tools derived from direct comparative analysis of microbial opsins,” Nat. Methods 9, 159–172 (2011).1548-7091 10.1038/nmeth.1808 22179551 PMC4165888

[16] Tanimura A. et al., “Cholinergic interneurons amplify corticostriatal synaptic responses in the Q175 model of huntington’s disease,” Front. Syst. Neurosci. 10, 102 (2016). 10.3389/fnsys.2016.00102 28018188 PMC5159611

[r17] Bock R. et al., “Strengthening the accumbal indirect pathway promotes resilience to compulsive cocaine use,” Nat. Neurosci. 16, 632–638 (2013). NANEFN 1097-6256 10.1038/nn.3369 23542690 PMC3637872

[r18] Kosillo P. et al., “Cortical control of striatal dopamine transmission via striatal cholinergic interneurons,” Cereb. Cortex 26, 4160–4169 (2016). 10.1093/cercor/bhw252 PMC5066833 27566978

[19] Mingote S. et al., “Functional connectome analysis of dopamine neuron glutamatergic connections in forebrain regions,” J. Neurosci. 35, 16259–16271 (2015). JNRSDS 0270-6474 10.1523/JNEUROSCI.1674-15.2015 26658874 PMC4682788

[20] Konieczny J. Lenda T. Czarnecka A., “Early increase in dopamine release in the ipsilateral striatum after unilateral intranigral administration of lactacystin produces spontaneous contralateral rotations in rats,” Neuroscience 324, 92–106 (2016). 10.1016/j.neuroscience.2016.02.072 26964686

[21] Bartholomew R. A. et al., “Striatonigral control of movement velocity in mice,” Eur. J. Neurosci. 43, 1097–1110 (2016). EJONEI 0953-816X 10.1111/ejn.13187 27091436

[22] Kravitz A. V. et al., “Regulation of parkinsonian motor behaviours by optogenetic control of basal ganglia circuitry,” Nature 466, 622–626 (2010). 10.1038/nature09159 20613723 PMC3552484

